# Complete analysis and phylogenetic analysis of *Polygonatum sibiricum* mitochondria

**DOI:** 10.1186/s12870-025-06510-0

**Published:** 2025-04-15

**Authors:** Min Liu, Ruike Fan, Chen Wang, Lishang Dai, Shenghui Chu

**Affiliations:** 1 Key Laboratory of Xinjiang Phytomedicine Resource and Utilization, Ministry of Education, School of Pharmacy, Shihezi University, Shihezi, 832003 P.R. China; 2 School of Traditional Chinese Medicine, Wenzhou Medical University, Wenzhou, 325035 P.R. China

**Keywords:** *Polygonatum sibiricum*, Mitogenome, Repeated sequences, Phylogenetic relationship

## Abstract

In this project, we studied the complete mitogenome of the liliaceae medicinal plant *Polygonatum sibiricum*. The genome is represented by a circular ring molecule with a length of 691*,*910 bp and a GC content of 46.33%. Mitochondrial genome composition is slightly biased towards A+T, with AT accounting for 53.67%, and AT skewness slightly positive (0.092%). The complete mitogenome has a total of sixty-three unique genes, including thirty-nine protein-coding genes, twenty-one transfer RNAs (tRNAs) and three ribosomal RNAs (rRNAs). We examined codon use, repeat sequence, RNA editing in the mitogenome of *P. sibiricum*, and elucidated species classification based on phylogenetic trees of mitogenome of twenty-three species. Our results provide comprehensive information on the mitogenome of *P. sibiricum* and show for the first time the evolutionary relationship between the mitogenome of *P. sibiricum* and *Chlorophytum comosum* in the Asparagales family.

**Supplementary Information:**

The online version contains supplementary material available at 10.1186/s12870-025-06510-0.

## Introduction

*Polygonatum sibiricum* is a plant in the Liliaceae*,* and the 2015 edition of the Chinese Pharmacopoeia records three commonly used medicinal *P. sibiricum* Delar*. ex* Redoute or *P. cyrtonema* Hua. It originated in the Hengduan Mountain-Himalayan region twenty million years ago, and then the genus has diversified due to crustal changes, global climate cooling/drought and increased winter winds in East Asia. Subsequently stronger east Asian summer winds and global warming promoted the spread of the genus to other regions. It is currently distributed in the temperate regions of the northern hemisphere, such as China, Japan, South Korea, India, and other regions. In China, *P. sibiricum* is mainly distributed in Sichuan, Guizhou, Hunan, Anhui, Zhejiang, Fujian and other places [1–3]. Most Polygonum species grow in moist and shady places, usually in dense or fertile soil of forests or shrubs. By exploring the origin and evolution process of *P. sibiricum*, the researchers found that *P. sibiricum* is a monophyletic group, divided into two clades, corresponding to the round leaf group, mutual leaf group and Polygonatum group. Wheorlleaves, milky white flowers and short tepcanons are ancestral traits of the genus *P. sibiricum*. The evolution of flower coat color reflects the species’ adaptation to pollinators in different regions. Karyotypic changes, reduced chromosome number aneuploidy and polyploidization processes within *P. sibiricum* promote species differentiation and adaptive evolution [4]. It is one of China’s traditional herbs and widely used in the research and development of functional food [5, 6]. Researchers have found that *P. sibiricum* has the effects of lowering blood sugar, protecting nerves, anti-inflammatory, antioxidant, anti-osteoporosis, anti-fatigue and enhancing immunity [2], and can be used to treat diabetes, neuropathic pain, inflammation and infection, loss of appetite and so on [7, 8]. However, up to now, most studies have focused on pharmacological activity, main active ingredients, and food use, but the studies on its molecular level are relatively few. To date, only studies on the chloroplast genome have been found, but none are available on mitochondrial genome studies [9]. Therefore, the research aim of this work was to explore the mitochondrial genome of *P. sibiricum* by comparison with other species of the Liliaceae. The sequencing, assembly, annotation, and analysis of mitochondrial genomes can not only provide important reference data for evolutionary studies, population genetics and molecular breeding of Liliaceae species, but also help us to understand the biological characteristics of Liliaceae and to further understand the biological characteristics of Liliaceae species. This provides a reference for the identification of genuine *P. sibiricum* products.

Mitochondria are two-layer membrane-covered organelles present in most eukaryotic cells, where energy is produced, and the cells perform aerobic respiration [10]. In the 1970 s, scientists discovered similarities between mitochondrial DNA in eukaryotes and bacterial genomes. Subsequent research indicated a close connection between mitochondria and α-proteobacteria that existed 1.5 billion years ago [11]. This relationship facilitated rapid evolution through multiple structural variations, recombination, and gene transfer. Compared with the mitogenome of animals and fungus, the mitogenome of plants differ in size, genome content and gene structure, ranging from tens and hundreds of thousands to several trillion bases [12, 13]. For example, *Picea satchensis* is the gymnosperm with the largest mitogenome found so far, with a size of 117 Mb, while *Viscum scurruloideum* is the smallest mitogenome found so far with only 66 kb [14, 15]. At the same time, mitogenome also shows a variety of gene content. In the study of *Viscum scurruloideum*, it was found that the gene content decreased with the decrease of the mitogenome, and this decrease may be due to the deficiency of NADH dehydrogenase, which is also seen in many angiosperms. In addition, the mitogenome of plants also produces the situation of untight gene distribution, gene deletion, DNA sequence transfer and so on*,* Therefore, the structure of the plant mitogenome is often considered mysterious [15, 16]. It has been reported that plant mitochondrial DNA is usually interconverted between an expert cycle configuration consisting of all genome sequences and a set of subgenomic cycles mediated by repetitions. However, the circular structure is not the only form of the plant mitogenome, there are many linear, multi-chromosome, and branching structures [17–19]. For example, the complete mitogenome of *Vitex rotundifolia* was mapped to a round molecule 380*,*980 bp long, the complete mitochondrial genome of *Prunus pedunculata* was mapped to a round molecule 405,855 bp long, and the *Quercus acutissima* was mapped to three sets of molecular structures, one linear molecule with a length of 224*,*233 bp. And two circular molecules 188*,*259 bp and 36*,*490 bp in length, respectively [20–22]*.* In recent years, researchers have characterized the size, structure and gene content of plant mitogenome, but there are still few relevant studies on plant mitogenome.

Therefore, we adopted the second and third-generation sequencing strategy to complete the assembly of the mitogenome of *P. sibiricum*. We characterized the size, structure, and gene content of the mitogenome. The mitogenome of *P. sibiricum* was further analyzed by displaying genomic features, repeat sequences, codon preference and RNA editing. The phylogenetic analysis of twenty-three angiosperm species by the genes present in the mitogenome further helps to reveal the inheritance of the mitogenome of *P. sibiricum*.

## Materials and methods

### Plant materials collection, DNA extraction, and sequencing

The fresh leaves of *P. sibiricum* were collected from Chizhou, Anhui Province and sent for sequencing. The person who formally identified *P. sibiricum* in this study is Associate Professor Zhigang Wu. The leaf specimens of the studied *P. sibiricum* have been in a publicly accessible herbarium, which is fully named as the Herbarium of Traditional Chinese Medicine, Wenzhou Medical University. The storage number is ‘HJ08’. Samples were frozen in liquid nitrogen and stored at − 80 ℃ until used. Total genomic DNA was extracted using a DNA secure Plant Kit according to the manufacturers protocol (TIANGEN, Beijing, China). The quality and concentration of the DNA products were assessed via agarose gel electrophoresis and spectrophotometry (NanoDrop- 2000, Thermo Fisher Scientific). The complete mitochondrial genomes and chloroplast genomes of *P. sibiricum* were obtained by the ‘3 + 2’ strategy which sequenced by the long-reads obtained from the Oxford Nanopore PromethION platform and corrected by the short-reads using the Illumina Novaseq 6000 platform [23]. A summary of the sequencing results of long-reads and short-reads were showed in Table S1 and Table S2, respectively.

In the second-generation sequencing experiment, DNA was fragmented by mechanical interruption (ultrasound), and then the whole library was prepared by fragment purification, end repair, polyA by 3’end addition, ligation of sequencing junction, and PCR amplification. The constructed libraries will be sequenced through the Illumina Novaseq6000 platform. The Sequenced Raw Reads was quality controlled by fastp (version 0.20.0, https://github.com/OpenGene/fastp) software to obtain clean Reads. Remove adapter in Reads and reads with Primer sequence and N number> 5; reads with average quality value <Q5 will be filtered.

The third-generation sequencing used single-molecule real-time (SMRT) sequencing technology [24]. The library was prepared by PacBio Binding kit, sheared DNA fragments with a main peak of approximately 15–18 kb were used, large fragments of DNA were enriched and purified using magnetic beads, and the fragmented DNA was end repaired. The stem-loop sequencing adaptors were then connected to both ends of the DNA fragment using the SQK-LSK109 ligation kit. The final constructed libraries were sequenced via the Oxford Nanopore PromethION platform, generated the raw data, and passed by filtlong (v0.2.1, https://link.zhihu.com/?target=https%3A//github.com/rrwick/Filtlong). The software filtered the third-generation sequencing data with the parameters: - -min_length 1000- -min_mean_q 7.

### Mitogenome assembly and annotation

The assembly strategy is as follows: 1). We utilized Minimap2 (v.2.24) to align the Nanopore reads to our draft assembly of *P. sibiricum* [25]. 2). The aligned reads were extracted and subjected to de novo assembly. 3). Initially, the original third-generation sequencing data were aligned to the seed sequence using minimap2, screened for sequences with overlap greater than 1 kb, added to the seed sequence, and iteratively aligned the original data to the seed sequence, thus obtaining all the third-generation sequencing data of the mitochondrial genome, and subsequently the resulting three-generation data were corrected using canu [26]. 4). After that, using Bowtie2 (v2.3.5.1) [27] to align the short-reads to the previous correction results, using Unicycler (v0.4.8) [28] for mixed assembly, and 5). Split the GFA file according to the coverage of the long reads to obtain the final assembly result.

The mitochondrial genomes were annotated by BlastN [29]. Mitochondrial genes were identified and queried against the NCBI database. Additionally, tRNA genes were detected using tRNA scan-SE software [30] (http://lowelab.ucsc.edu/tRNAscan-SE/); OpenReading Frame Finder (http://www.ncbi.nlm.nih.gov/gorf/gorf.html) software was used to annotate the ORF and annotate the nr libraries over three hundred; RNA editing sites were predicted using the PmtREP (http://112.86.217.82:9919/#/tool/alltool/detail/336) software. The boundaries of the introns were manually reviewed and corrected to ensure the complete structure of the protein-coding genes. The newly sequenced mitochondrial genomes were deposited in GenBank under the accession numbers PQ932596. Mitochondrial genome maps were constructed using the OGDRAW (https://chlorobox.mpimp-golm.mpg.de/OGDraw.html) [31]. The assembly procedure is described in the Supplementary Material Figure S1.

### Analysis of repeat sequences

We used trf software (trf409. linux64*,* parameters:2 7 7 80 10 50 2000 -f -d -m) to identify tandem*,* next*,* simple repeats were identified using misa software (v1.0*,*

parameters: 1–10 2–5 3–4 4 - 3 5 - 3 6 - 3) and dispersed was identified with blastn (v2.10. 1, parameter:-word_size 7, evalue 1e- 5, redundancy removed, tandem duplication removed) software, visualized with circos v0.69 - 5 and obtained results.

### RSCU analysis

Due to the degeneracy of codons, each amino acid corresponds to a minimum of one codon and a maximum of six codons. There are great differences in the utilization of genome codons in varied species and different organisms. This inequality in the use of synonymous codons is called codon bias. Use our own Perl script to filter the uniq CDS and do the calculations.

### RNA editing predicting

The principle of RNA editing site prediction in the mitogenome of *P. sibiricum* is based on multi-sequence alignment. The amino acid sequence of the target sequence is compared with multiple sequences in the database, and then the changes of each site of the target sequence in the corresponding position in the database are counted. If the conditions for RNA editing are met, this site may be a potential RNA editing site. So, we adopt PmtREP (http://112.86.217.82:9919/#/tool/alltool/detail/336) to forecast the yellow fine mitogenome RNA editing sites prediction.

### Phylogenetic analysis

In order to reveal the phylogeny of *P. sibiricum*, 23 angiosperm mitogenomes (Table S9) were selected from the NCBI Organelle Genome Resource database (https://www.ncbi.nlm.nih.gov/genome/browse/#!/organelles/). PhyloSuite (v.1.2.2) was used for extracting the common PCGs among the mitogenomes of the selected species sequences [32]. MAFFT (v7.471) was utilized for aligning the extracted sequences [33]. The maximum likelihood (ML) method implemented in RAxML v.8.2.4 The parameters were ‘raxmlHPC-PTHREADS-SSE3 -f a -N 1000 -m GTRGAMMA—× 551,314,260 -p 551,314,260’. The bootstrap analysis was performed with 1,000 replicates. Bayesian inferences (BI) analysis was performed by MrBayes (v3.2.7) with the GTR + G + I model and 1000 bootstrap replicates.

## Results

### Mitogenome assembly and annotation results

**Fig. 1:**
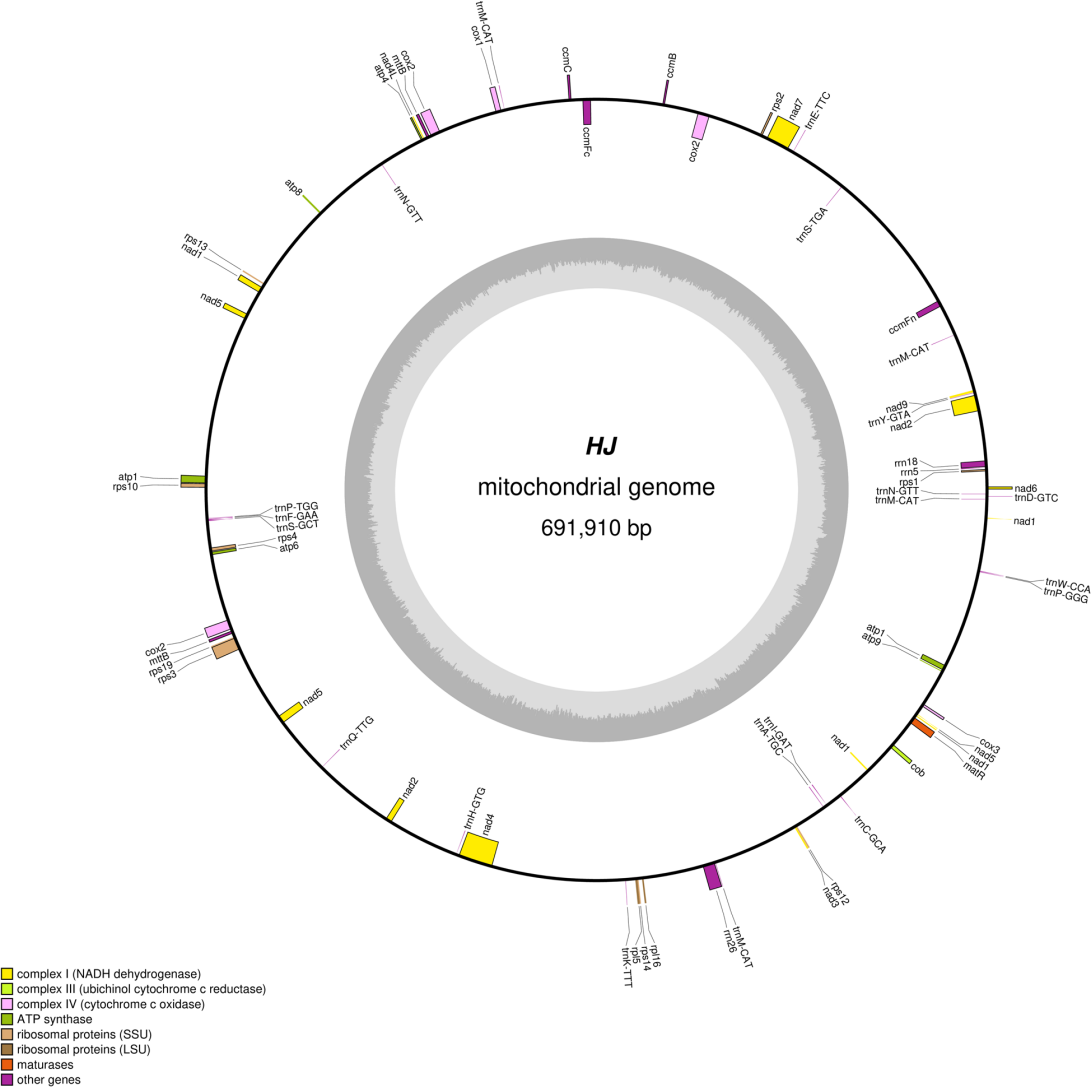
Schematic of the mitogenome of *P. sibiricum*. Genes on the inside are on the negative strand, and genes on the outside are on the positive strand. The inner grey circle shows the GC contents of the mitogenome. The circle inside the GC content graph shows the 50% threshold. The colors of genes show different functional categories, and details are shown in the legend. Genes with introns were marked with (*)

The mitogenome HJ- 1 of *P. sibiricum* was assembled into a circular molecule 691*,*910 bp long with a GC content of 46.33% (Fig. 1). There are sixty-three unique genes, including thirty-nine protein-coding genes and twenty-four non-protein-coding genes (Tables 1 and 2). And the total length of protein-coding genes was 35*,*691 bp, accounting for 5.16% of the total length of the genome. The mitogenome of *P. sibiricum* contains 14 core genes, respectively 5 ATP synthase genes (*atp1, atp4, atp6, atp8, atp9*)*,* 4 cytochrome c biogenesis genes (*ccmB, ccmC, ccmFc, ccmFn*)*,* 3 cytochrome c oxidase genes (*cox1, cox2, cox3*)*,* a ubiquinol cytochrome c reductase gene (*cob*) and a maturases gene (*matR*); Next*,* there were 21 variable genes, including 9 NADH dehydrogenase genes (*nad1, nad2, nad3, nad4, nad4L, nad5, nad6, nad7, nad9*)*,* 2 LSU (*rpl16, rpl5*)*,* 3 SSU (*rps1, rps10, rps12, rps13, rps14, rps19, rps2, rps3, rps4*)*,* 1 transport membrane protein gene (*mttB*) (Table S3)*,* No gene of succinate dehydrogenase was found. Then*,* there are eighteen transfer RNA (tRNA) genes and three ribosomal RNA (rRNA) genes (*rrn18, rrn26 and rrn5*). A total of twelve genes containing introns were identified, such as *nad1, nad2, nad5, nad7* with four introns. *nad4* with three introns, *cox2* with two introns, *ccmFc, rps10, rps3, trnA-TGC, trnI-GAT, trnN-GTT* with one intron, and four genes with multiple copies were found. Examples include two copies of *atp1, mttB*, three copies of *cox2*, and four copies of *trnM-CAT*.

**Table 1:** Genes predicted in the mitogenomes of *P. sibiricum*

Group of genes	Gene name
ATP synthase	atp1(2) atp4 atp6 atp8 atp9
Cytohrome c biogenesis	ccmB ccmC ccmFc* ccmFn
Complex III(Ubichinol cytochrome c reductase)	cob
Complex IV(Cytochrome c oxidase)	cox1 cox2**(3) cox3
Maturases	matR
Transport membrance protein	mttB(2)
Complex I(NADH dehydrogenase)	nad1**** nad2**** nad3 nad4*** nad4L nad5**** nad6 nad7**** nad9
Ribosomal proteins (LSU)	rpl16 rpl5
Ribosomal proteins (SSU)	rps1 rps10* rps12 rps13 rps14 rps19 rps2 rps3* rps4
Ribosomal RNAs	rrn18 rrn26 rrn5
Transfer RNAs	trnA-TGC* trnC-GCA trnD-GTC trnE-TTC trnF-GAA trnH-GTG trnI-GAT* trnK-TTT trnM-CAT(4) trnN-GTT trnN-GTT* trnP-GGG trnP-TGG trnQ-TTG trnS-GCT trnS-TGA trnW-CCA trnY-GTA

^*^intron number (*The number of asterisks denotes the quantity of introns: * (one intron), ** (two introns), *** (three introns), **** (four introns); Gene(2) Number of copies of multi-copy genes

**Table 2:** Mitogenomes content of *P. sibiricum*

Feature	GC(%)	Size(bp)	Proportion in genome(%)
genome	46.33	691910	100%
Protein-coding gene	43.78	35691	5.16%
tRNA	50.54	1571	0.23%
rRNA	53.3	5330	0.77%

**Fig. 2:**
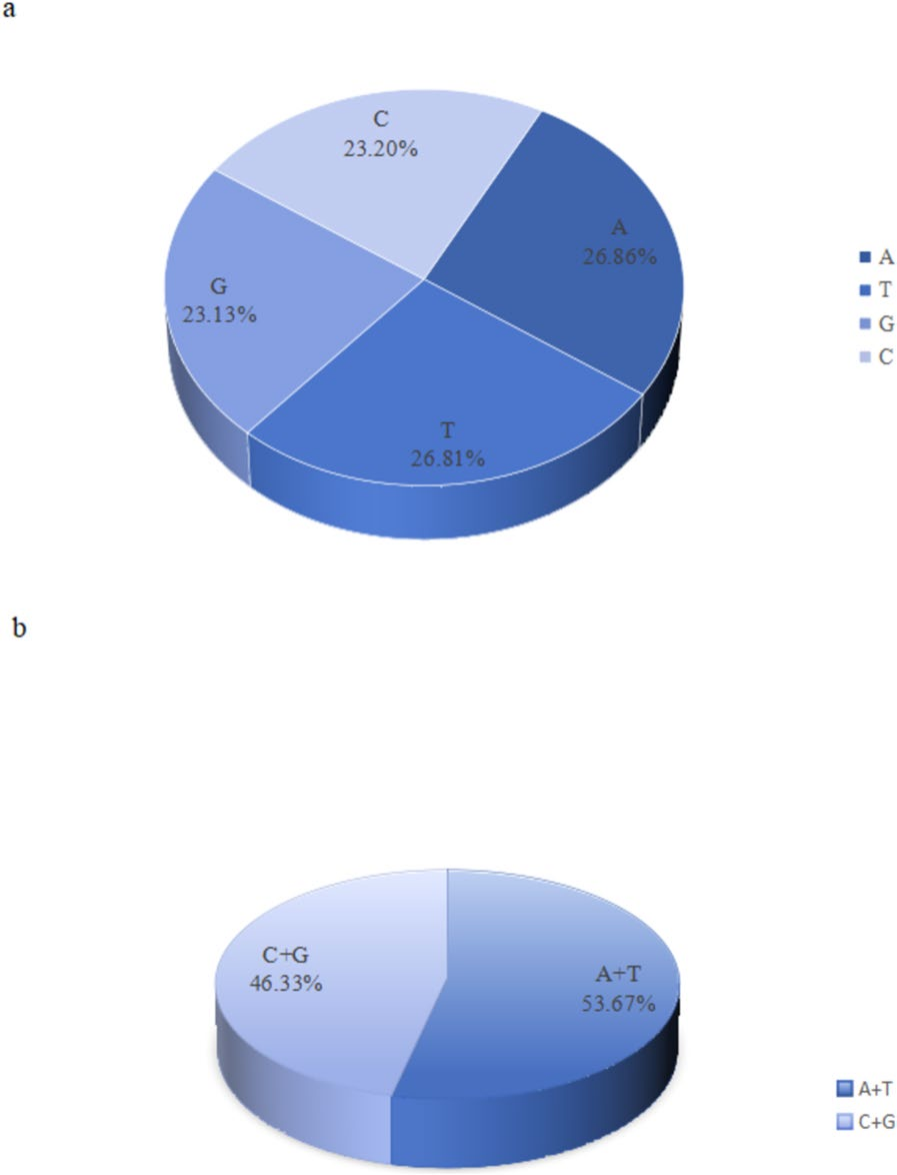
Composition in the *P. sibiricum* mitogenomes. **a** A, T, C, G four base proportions. **b** The proportion of A+T, C+G

At the same time, the analysis of the mitogenome showed that its nucleotide composition was biased towards As and Ts (A= 26.86%, T= 26.81%, C= 23.20%, G= 23.13%), and the total content of A+T and C+G in the mitogenome was 53.67% and 46.33% (Fig. 2), respectively. The base composition of a nucleotide sequence can be described by its skewness, that is, measured by the relative numbers of As to Ts and Gs to Cs. Through calculation, we found that the AT skewness of the mitogenome was slightly positive (0.092%), indicating that the number of A was more than T; the GC skewness was slightly negative (− 0.141%), indicating that the number of C was more than G.

### Repeat sequence in the mitogenome of *P. sibiricum*

**Fig. 3:**
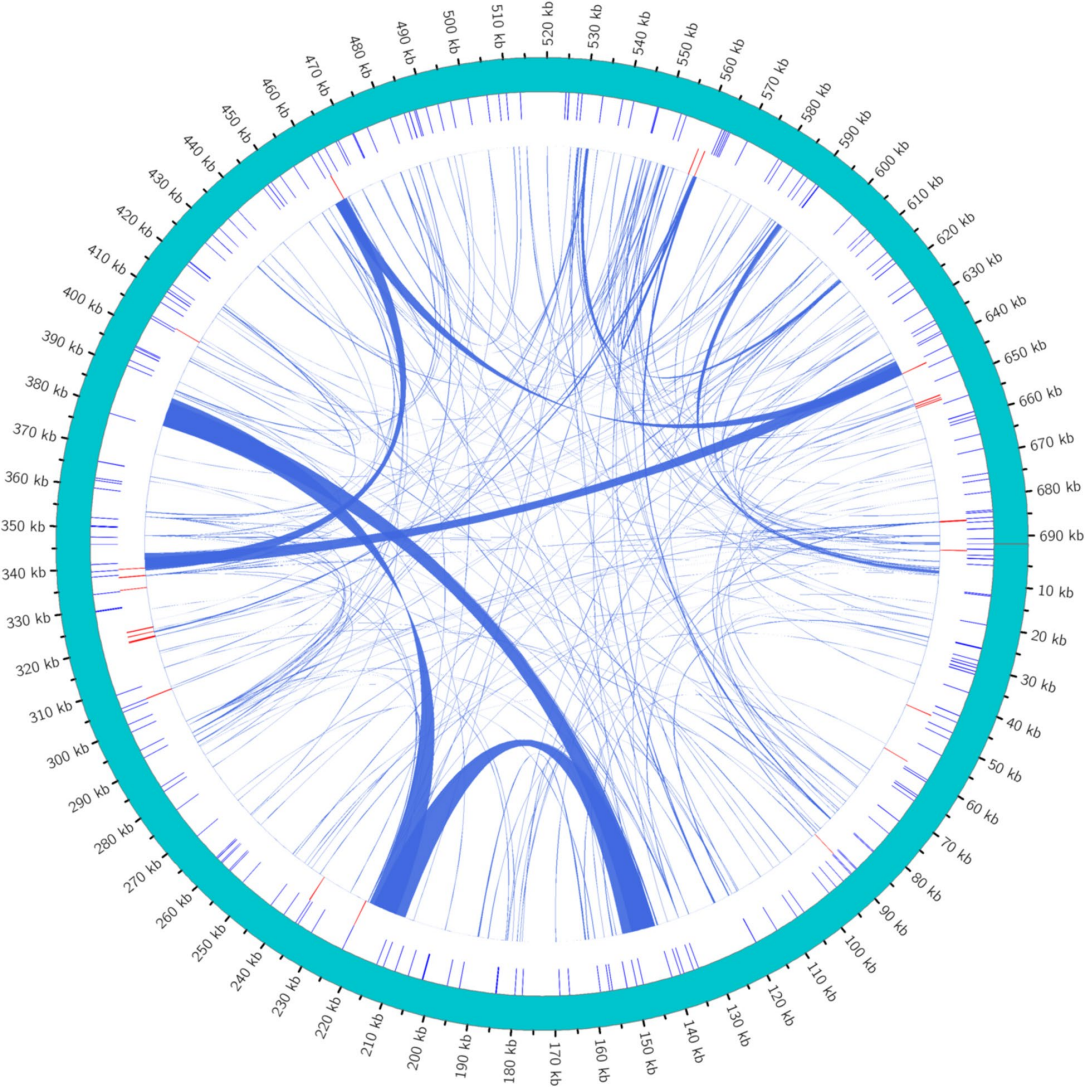
Map of the distribution of repeated sequences across the genome. The outermost ring represents the mitochondrial genome sequence, and the inner sequence is simple repeat sequence, tandem repeat sequence, and scattered repeat sequence

Repeat sequence includes SSR, tandem repeat sequence and scattered repeat sequence. Through identification of the *P. sibiricum* extract repeat sequence, the results were visualized by circos v0.69 - 5 software, and the results as shown in Fig. 3 were obtained. As can be seen from the figure, the length of repeat sequences in the genome of *P. sibiricum* ranges from 0 to 690 kb, and most of them are simple repeat sequences and scattered repeat sequences, with only a few simple repeat sequences.

**Fig. 4:**
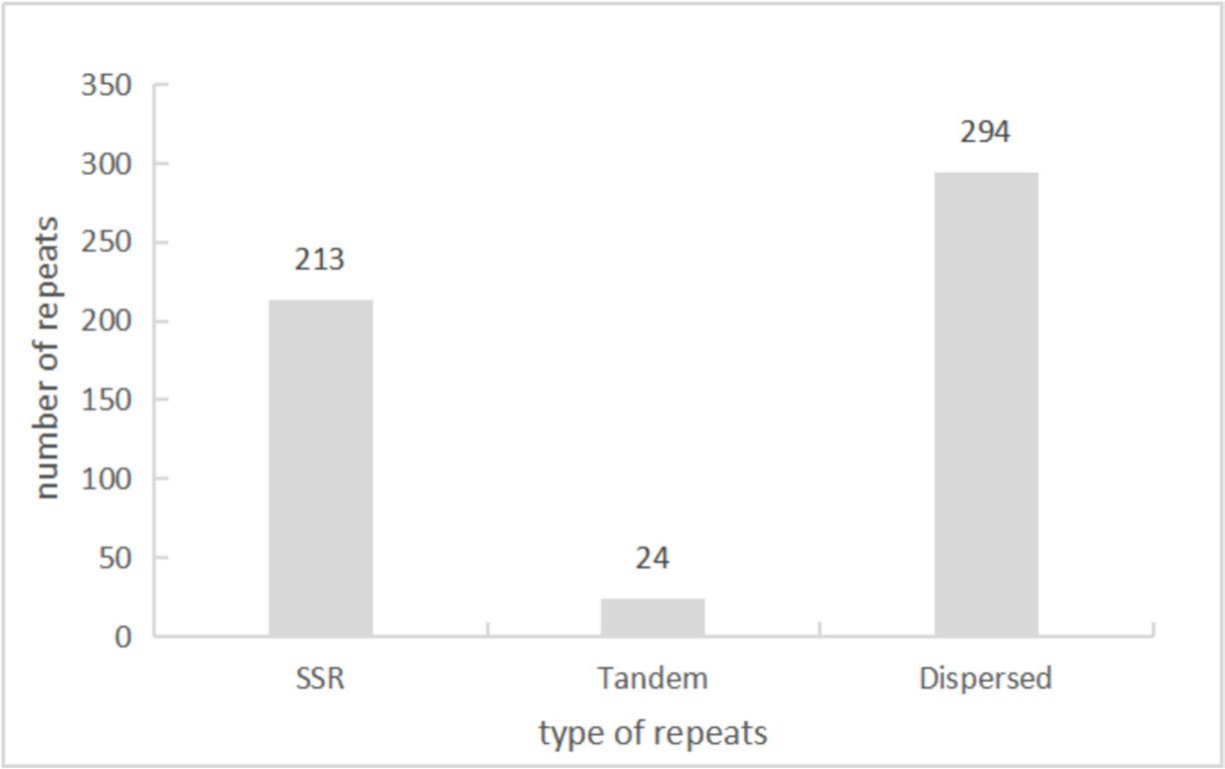
Distribution of repeat sequences. The x-axis represents the type of repeats, while the y-axis represents the number of repeats

**Fig. 5:**
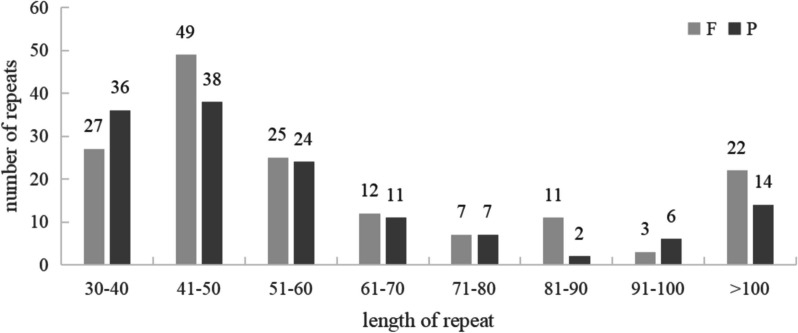
Distributed repeat sequence length allocation. The x-axis displays the length of repeats, and the y-axis displays the number of repeats

According to the detection results, we found 213 SSRs, twenty-four tandem repeats and 294 scattered repeats, which accounted for 40.1%, 4.5% and 55.4% of the total repeats, respectively (Fig. 4). There were 138 palindromic repeats and 156 forward repeats, accounting for 53.1% and 46.9% of the total. The length distribution of repeated sequences was uneven, mostly concentrated in the range of 30–100 bp, accounting for 87.8% of the total number, and thirty-six sequences exceeding 100 bp, accounting for 12.2% of the total number, among which the largest sequence length could reach 9,217 bp (Fig. 5).

**Fig. 6:**
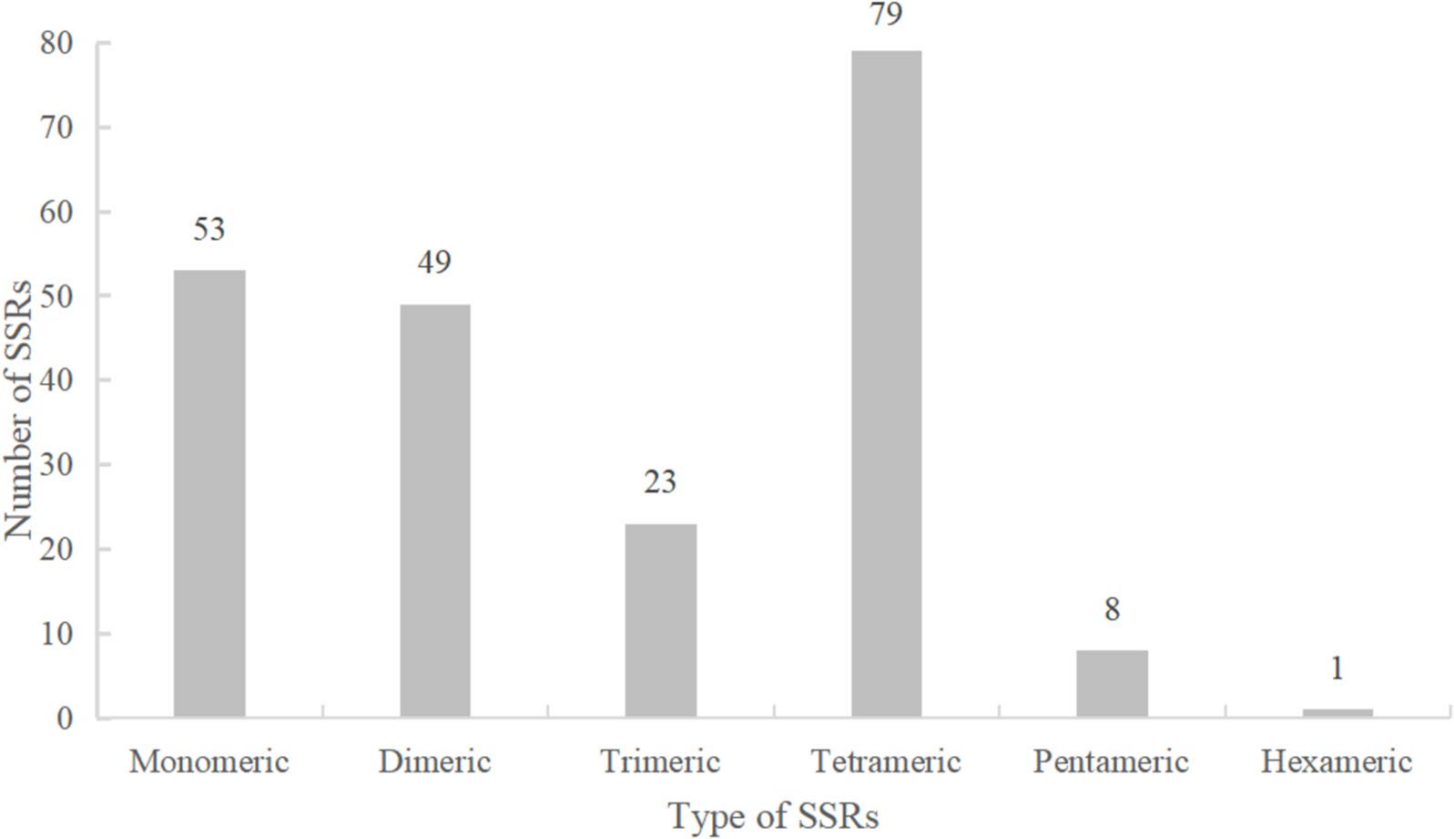
Nucleotide repeat unit of *P. sibiricum* mitogenomes. The x-axis represents the type of SSRs while the y-axis represents the number of repeats

For SSR, we found that tetramers accounted for the largest proportion (37.1%), up to seventy-nine, followed by monomers (27.2%) and dimers (23.0%), 58 and 49, respectively, while hexamers accounted for the least, only 1 (Fig. 6). Among all SSRs, only four repeat sequences did not contain A/T, and the other 209 sequences all contained A/T. Of these 209 sequences, 68 SSRs were found to consist entirely of A/T, including fifty monomers (A/T), ten dimers (AT/TA), three trimers (AAT/ATT), and five tetramers (AAAT/ATTT, AATT/AATT). The A/T richness of 132 SSRs ranged from 50% to 83% (Table S4).

In addition, *P. sibiricum* in the mitogenome with long 15–42 bp of tandem repeat sequences, matching degree were over 80%, and have a copy, including CAGGGATTTCATCGGGCTGTAGCTATGCGGCTA sequence repeat number up to 6.4 times (Table S5).

### Analysis of codon usage in *P. sibiricum*

**Fig. 7:**
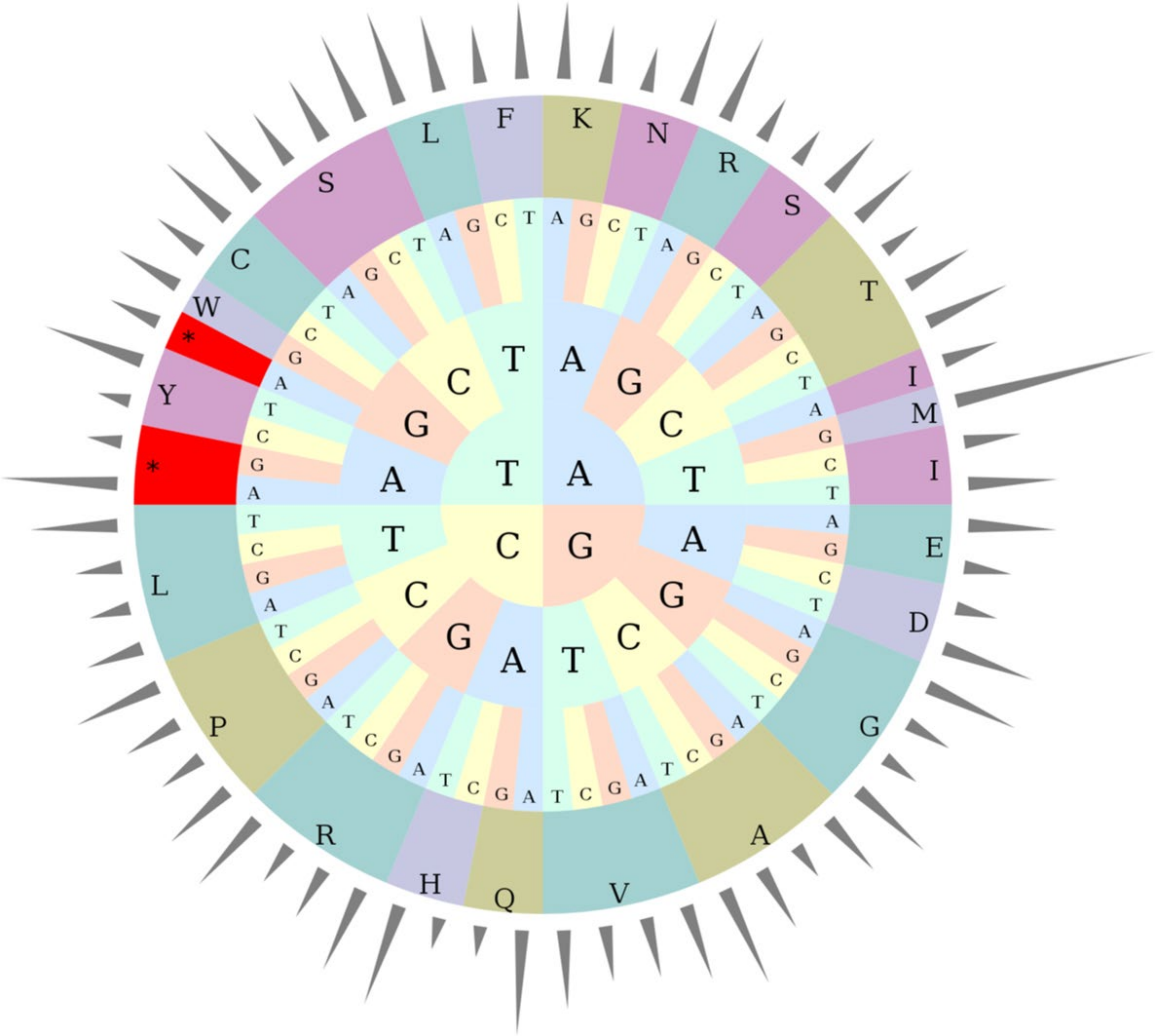
Pie chart of RSCU. The height of the outermost cylinder is the RSCU value, the inner layer is the amino acid, the innermost three layers are the codon

**Fig. 8:**
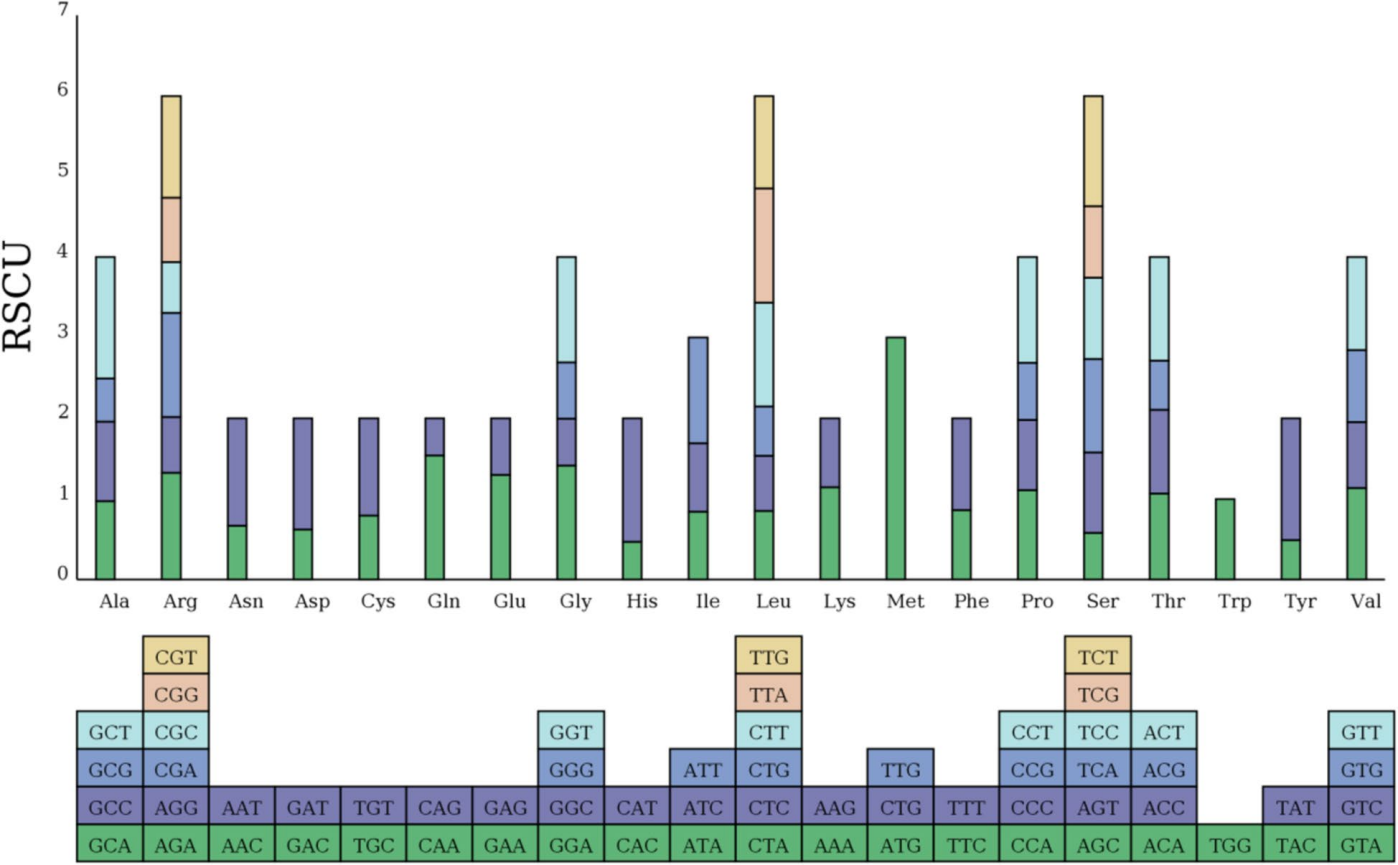
The synonymous codon of the mitogenomes of *P. sibiricum* was used. X-axis, codon families; Y-axis, the relative synonymous codon usage (RSCU) value. RSCU measures the likelihood of a specific codon being used among synonymous codons that encode the same amino acid and values greater than one indicate a higher frequency of usage for the codon

The organism contains a total of sixty-six universal codons that occur 10,589 times in the mitochondrial genome (Table 3), encoding more than twenty different proteins. Among the various amino acids, only Met and Trp are composed by single-species codons (Fig. 7), and all other amino acids are composed by multiple codons (Fig. 8), like other angiosperm genomes. Synonymous codons are the same amino acids used to encode different sequences, reflecting the codon degradation situation, and their third basis is usually different. We usually employ RSCU to judge the frequency of synonymous codons in biological translation. It was found that the most common codon in the mitochondrial genome is AUG with an RSCU value of 3.00 and the highest proportion of mitochondria. Then was the UAA encoding the stop codon, with an RSCU value of 1.71, and in the third place was the CAA encoding Gln, with an RSCU value of 1.54. A total of thirty-three codons were detected with RSCU values greater than 1.00 (Table S6), indicating that the high frequency of use of these codons. Meanwhile, out of the thirty-three codons, twenty-eight were found to end with A/T (U) bases, including 12 A bases and 16 T bases, indicating the A/T preference of the usage patterns of these codons.

**Table 3:** Mitogenomes codon count of *P. sibiricum*

CODON	COUNT	CODON	COUNT	CODON	COUNT
UAA(*)	20	AUC(I)	228	AGG(R)	86
UAG(*)	6	AUU(I)	354	CGA(R)	162
UGA(*)	9	AAA(K)	249	CGC(R)	79
GCA(A)	162	AAG(K)	186	CGG(R)	100
GCC(A)	163	CUA(L)	155	CGU(R)	158
GCG(A)	89	CUC(L)	125	AGC(S)	97
GCU(A)	250	CUG(L)	111	AGU(S)	168
UGC(C)	56	CUU(L)	234	UCA(S)	194
UGU(C)	85	UUA(L)	258	UCC(S)	169
GAC(D)	104	UUG(L)	209	UCG(S)	149
GAU(D)	231	AUG(M)	287	UCU(S)	230
GAA(E)	295	CUG(M)	0	ACA(T)	145
GAG(E)	159	UUG(M)	0	ACC(T)	141
UUC(F)	292	AAC(N)	111	ACG(T)	83
UUU(F)	383	AAU(N)	221	ACU(T)	175
GGA(G)	261	CCA(P)	176	GUA(V)	185
GGC(G)	106	CCC(P)	137	GUC(V)	133
GGG(G)	129	CCG(P)	112	GUG(V)	145
GGU(G)	241	CCU(P)	208	GUU(V)	188
CAC(H)	63	CAA(Q)	219	UGG(W)	144
CAU(H)	204	CAG(Q)	65	UAC(Y)	76
AUA(I)	228	AGA(R)	166	UAU(Y)	235

Note: *: the termination codon

### RNA editing result

**Fig. 9:**
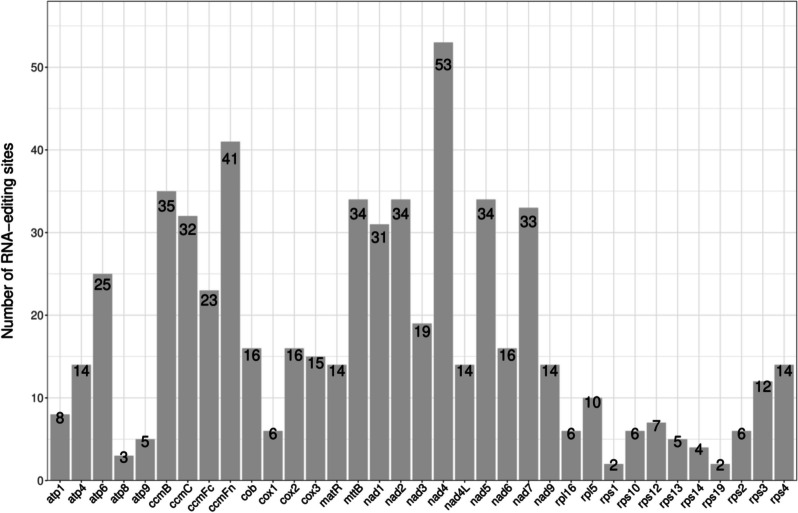
RNA editing sites in protein-coding gene sequence of the mitogenomes of *P. sibiricum.* The x-axis displays the gene, and the y-axis displays the number

Six hundred nine RNA editing sites were identified from thirty-nine protein-coding genes in the mitogenome of *P. sibiricum*, all of which were C-to-U RNA editing. Most of the predicted RNA editing occurred at the first (34.3%) and second (62.2%) sites, but some editing sites had changes at both the first and second sites (3.4%). At the same time, it was found that NADH dehydrogenase had the largest number of RNA editing sites (248), among which *nad4* gene editing accounted for the largest number of RNA sites in the total mitochondrial genome, up to fifty-three. In addition, only 2 RNA editing sites were predicted by *rps1* and *rps19* in the whole mitochondria (Fig. 9), and RNA editing sites existed in all genes. A total of fourteen amino acid transitions were identified at these RNA editing sites. Among all identified mutations, the most common amino acid changes were serine (S) to leucine (L) and proline (P) to leucine (L) and, with a frequency of 128 and 123 times, respectively (Table S7). In addition, through RNA editing, we found that 42.86% of the amino acids retained the original hydrophobic or hydrophilic, 47.29% of the amino acids changed from hydrophilic to hydrophobic, and 9.20% of the hydrophobic amino acids changed to hydrophilic (Table S8). In addition, 0.66% of the editing sites lead to stop-coding, and these genes are in *atp 6, atp 9, ccmFc*, and *rps 10*. This finding indicates that hydrophilic amino acids can change towards hydrophobic amino acids, which will help to improve the protein stability in the mitochondrial genome of *P. sibiricum.*

### Phylogenetic analysis

**Fig. 10:**
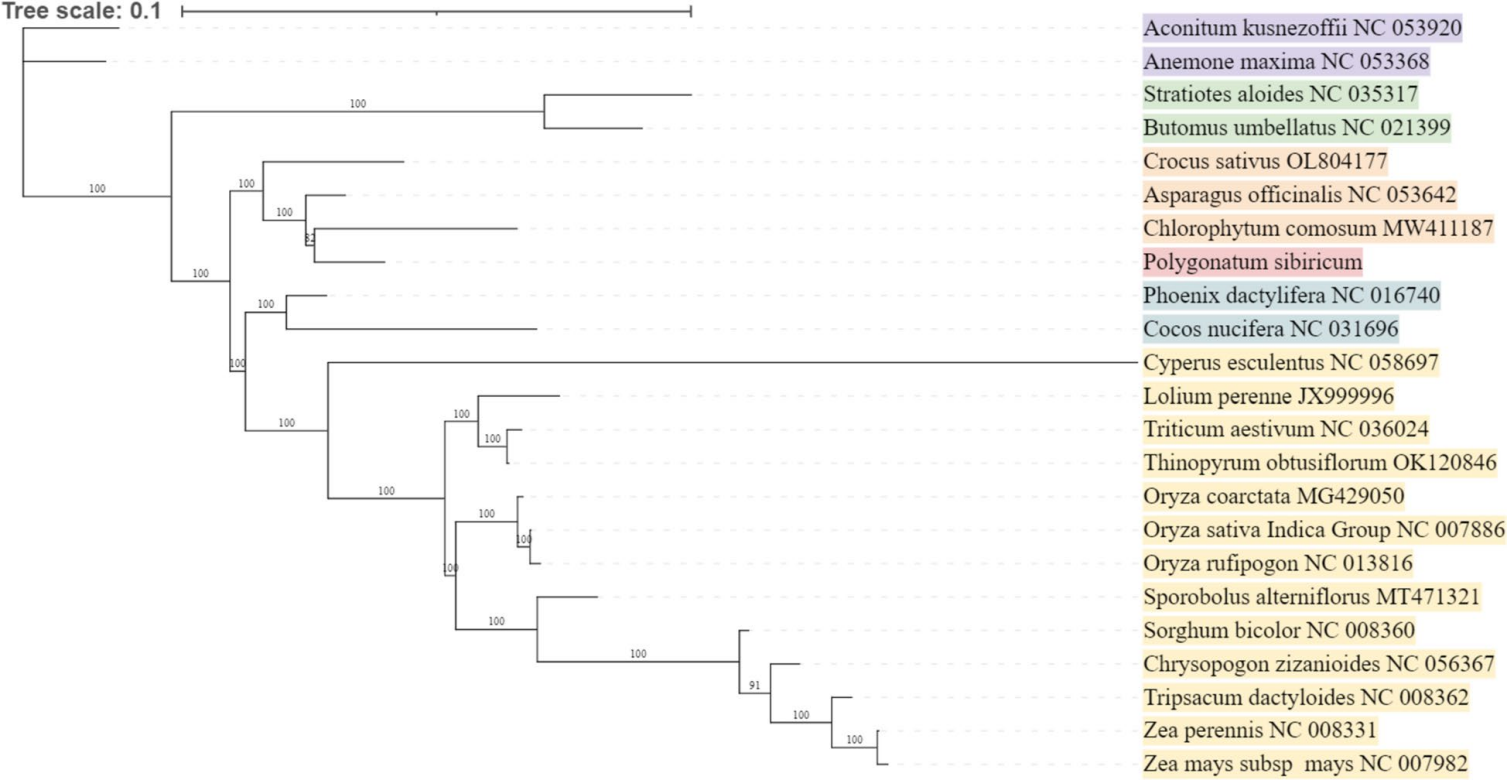
Phylogenetic tree of 23 angiosperm based on the sequences of conserved mitochondrial PCGs. Phylogenetic tree of 23 angiosperm based on the sequences of conserved mitochondrial PCGs. The ranunculales were chosen as the outgroup

Because there are few mitochondrial genome sequences in Liliaceae and Lilium species, we constructed a phylogenetic tree from Asparagales, Arecales, Alismatales, and Poales based on sequence divergence analysis and based on mitochondrial PCG conserved sequence, with Ranunculales as the foreign species (Fig. 10). The results show that the *Chlorophytum comosum* of Asparagales are closely related, which is consistent with the previous idea of both Liliaceae and Asparidae, which further proves that the mitochondrial genome can provide evidence for the genetic evolution of plants.

## Discussion

Mitochondria are the sites of energy metabolism of eukaryotic cells and provide energy for the life activities of various cells. In plants, mitogenome profoundly affect their growth and development, chloroplast function and cross-compatibility [34]. Compared with humans and animals, the most significant feature of plant mitochondria is that mtDNA is large and can rapidly evolve structurally. In addition, the mtDNA of land plants can reach 200–400 kb, compared with 16.6 kb of circular mtDNA of humans. But despite this, the mitogenome of plants still encodes only a few genes, only about twenty more than that of animals and yeast. Arabidopsis, for example, has 367 kb of mtDNA but encodes only 32 proteins, 22 tRNAs, and 3 rRNAs [35]. In addition, compared with animal mitochondria, the coding sequences of plant mitochondria are more complex and diverse, and there are often situations such as size variation, sequence alignment, duplicate content and high conservation [19, 36]. In this study, the total mtDNA length of *P. sibiricum* was 691,910 bp and GC content was 46.33%, while the Rice bean mtDNA was 404,493 bp, GC content was 45.18% [37], *Camellia drupifera* was 970,986 bp, GC content was 45.73% [38], and *Calla Lilies* mtDNA was 675,575 bp and GC content was 45.85% [39]. The mtDNA of *P. sibiricum* is intermediate in size but has high GC content. Combined with the differences in the number of encoded proteins of the three class species, it is speculated that the gene differences of encoded functional proteins may require higher GC content of the mtDNA to ensure the stability of gene function.

Repeat sequences in the mtDNA are responsible for rearrangements and size changes by homologous recombination. High recombination activity means that gene order varies even at the level of specificity. Repeats in mitogenome are often critical for intermolecular recombination, and in general, the largest repeats within a species (typically more than about 1 kb in angiosperms) are found to occur leading to is omerization [39, 40]. The longest dispersal repeat in the mitochondrial genome of *P. sibiricum* exceeds 1 kb (9,217 bp in size), which may be the cause of heterodimerization. In addition, we studied the repeat composition of the three plant species and found that there were 192 SSRs in *Fritillaria ussuriensis*, with the largest tetramers of 53.65%, did not find the presence of hexamers, and in 37 monomers, 97.3% of the sequences were A/T repeats [41]. In Theaceae, *Camellia drupifera* found 269 SSRs, the largest tetramers, which was 40.15%. While the least was hexamers, 1.48% [38]. However, in the *P. sibiricum*, we found 213 SSRs, 37.1% tetramers, the least hexamer content, only one, in these SSRs, 98.12% of the sequences were A/T repeats. Since the total number of SSRs is weakly correlated with the size of the mtDNA, it is considered that the increase of repeats may not significantly promote genome expansion.

To explore the codon usage of PCGs, we analyzed the codon distribution and relative synonymous codon usage (RSCU) frequency of *P. sibiricum*, and found that, like most resolved plant mitochondria, *P. sibiricum* showed a general preference for Leu and Ser in amino acid utilization, while showing the least expression in Trp and Cys. Codons with an RSCU greater than one are defined as high-frequency codons and are considered to be preferentially used in amino acid coding. In *P. sibiricum* mitochondria, most of the amino acids except for UGG and AUG. A total of thirty-three high-frequency codons were identified in the *P. sibiricum* mitochondria, indicating a high preference for the usage of these codons. The RSCU values of UAA (termination), GCU (Ala), CAU (His), AUG (Met), CAA (Gln) and UAU (Tyr) were greater than 1.5, showing a high degree of codon usage preference. In addition, by comparing with the *Lilium tsingtauense* of Liliaceae and *Populus tomentosa* of Salicaceae [42, 43], we found that there is A universal codon preference of A or T (U) in the third position of high frequency codons in the *P. sibiricum*, indicating that different genome and genome codons may have similar preferences in different species.

In plants, gene expression requires RNA editing, and cytidine (C) to uridine (U) RNA editing is abundant in mitochondrial and chloroplast genomes [44]. Studying RNA editing sites helps to understand the expression patterns of mitochondrial and chloroplast genes in plants, and thus to infer the state of evolution [45, 46]. Previous studies have identified approximately 491 RNA editing sites in 34 genes in *Maize NB* [47], 486 RNA editing sites in 31 genes in *Common Bean* [48], and 421 RNA editing sites in 26 genes in *Acer truncatum* [44]. This evidence suggests that genes of these categories are more sensitive to RNA modifications. In this study, 466 RNA editing sites were identified in 32 PCGs based on online site predictions, all of which exhibited CU RNA editing and all of which varied at 1, 2-bit codons, consistent with Sloan et al [49]. The number of RNA editing sites varies widely among different genes, but cytochrome c biogenesis and NADH dehydrogenase genes are the most numerous. Moreover, the analysis found that the amino acids in the codons of the *P. sibiricum* editing site showed a trend of Leu formation, accounting for 41.2% of the total amino acid transition, and more than 40% of the hydrophilic amino acids changed into hydrophobic amino acids, which is similar to the *garden asparagus* change in Liliaceae [50]. The amino acid changes produced by RNA editing contribute to the effects of physicochemical modifications on proteins, which in turn alter protein stability.

Meanwhile, phylogenetic tree was constructed based on mitogenome information to further analyze the phylogenetic relationship of *P. sibiricum*, and it was found that flavin was closely related to *Chlorophytum comosum*.

## Conclusions

In this study, the *P. sibiricum* mitochondrial genome was assembled into a circular molecule with a length of 691*,*910 bp, encoding a total of sixty-three genes, including thirty-five polynuclear genomes, three rRNAs, and eighteen tRNAs. The genome contains 213 SSRs, twenty-four tandem repeats, and 294 scattered repeats. At the same time, the use of *P. sibiricum* precision code, RNA editing and phylogenetic relationship were further analyzed. In conclusion, understanding the mitochondrial genome of *P. sibiricum* is of great significance to promote the understanding of the evolution of genetic background and provides a basis for genetic breeding.

## Supplementary Information

Supplementary Material 1.

Supplementary Material 2.

Supplementary Material 3.

## Data Availability

Sequence data that support the findings of this study have been deposited in the SRA at https://www.ncbi.nlm.nih.gov/sra/, reference number SRR29773581.
