# Supplementary figures and images for: Complete analysis and phylogenetic analysis of Polygonatum sibiricum mitochondria
Source: BMC Plant Biol. 2025 Apr 15;25:471. doi: 10.1186/s12870-025-06510-0 (PMC11998138; doi:10.1186/s12870-025-06510-0)

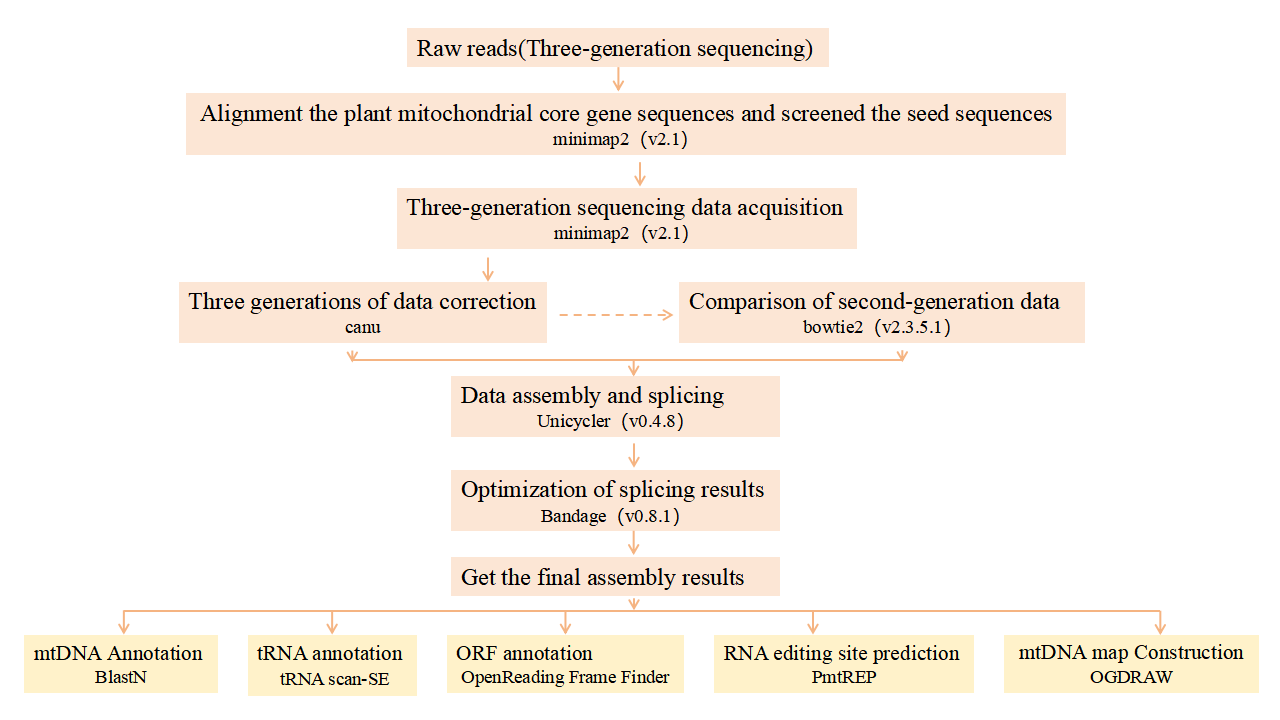

Supplement: Supplementary file 2 — Supplementary Material 2. [file 12870_2025_6510_MOESM2_ESM.tif]

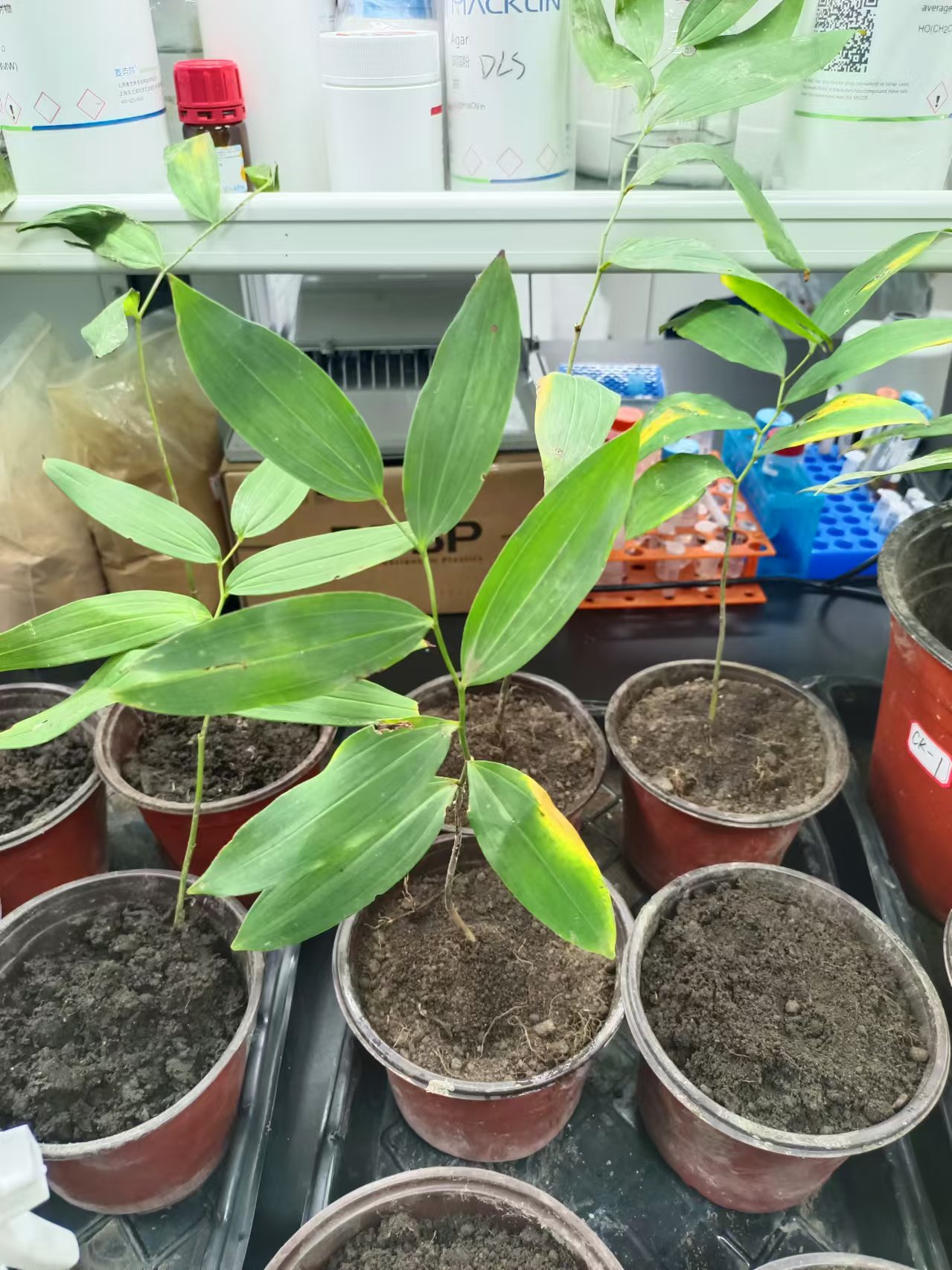

Supplement: Supplementary file 3 — Supplementary Material 3. [file 12870_2025_6510_MOESM3_ESM.tif]
